# Sickle cell disease: wheeze or asthma?

**DOI:** 10.1186/s40733-015-0014-2

**Published:** 2015-12-08

**Authors:** Robyn T. Cohen, Elizabeth S. Klings, Robert C. Strunk

**Affiliations:** 1 Division of Pediatric Pulmonary and Allergy, Department of Pediatrics, Boston University School of Medicine, 850 Harrison Avenue, Boston, MA 02118 USA; 2 Pulmonary Center, Department of Medicine, Boston University School of Medicine, 72 East Concord Street, Boston, MA 02118 USA; 3 Division of Allergy, Immunology, and Pulmonary Medicine, Department of Pediatrics, Washington University School of Medicine, 660 South Euclid Avenue, St. Louis, MO USA

**Keywords:** Sickle cell disease, Asthma, Wheezing, Airway obstruction, Airway hyperresponsiveness, Prevalence, Lung function

## Abstract

Sickle cell disease (SCD) is the most common life-limiting genetic disease among African Americans, affecting more than 100,000 people in the United States. Respiratory disorders in patients with sickle cell disease have been associated with increased morbidity and mortality. Associations between asthma and pain, acute chest syndrome (ACS), and even death have long been reported. More recently wheezing, even in the absence of an asthma diagnosis, has gained attention as a possible marker of SCD severity. Several challenges exist with regards to making the diagnosis of asthma in patients with SCD, including the high prevalence of wheezing, evidence of airway obstruction on pulmonary function testing, and/or airway hyperresponsiveness among patients with SCD. These features often occur in isolation, in the absence of other clinical criteria necessary for an asthma diagnosis. In this review we will summarize: 1) Our current understanding of the epidemiology of asthma, wheezing, airway obstruction, and airway responsiveness among patients with SCD; 2) The evidence supporting associations with SCD morbidity; 3) Our understanding of the pathophysiology of airway inflammation in SCD; 4) Current approaches to diagnosis and management of asthma in SCD; and 5) Future directions.

## Background

Sickle cell disease (SCD) is the most common life-limiting genetic disease among African Americans, affecting more than 100,000 people in the United States [1]. Respiratory disorders in patients with sickle cell disease have been associated with increased morbidity and mortality. Associations between asthma and pain, acute chest syndrome (ACS), and even death have long been reported. More recently wheezing, even in the absence of an asthma diagnosis, has gained attention as a possible marker of SCD severity. In this review we will summarize: 1) Our current understanding of the epidemiology of asthma, wheezing, airway obstruction, and airway responsiveness among patients with sickle cell disease; 2) The evidence supporting associations with SCD morbidity; 3) Our understanding of the pathophysiology of airway inflammation in SCD; 4) Current treatment options; and 5) Future directions.

### Asthma in SCD: why all the fuss?

Asthma is the most common chronic disease of childhood, and African Americans are disproportionately affected. Recent US data demonstrate an asthma prevalence of 15 % among African American children versus 8 % among Caucasian children [2]. Thus, we can extrapolate that a substantial percentage of children with SCD are going to have asthma. In the past decade, there have been studies suggesting that 1) asthma occurs at a similar frequency among African Americans with and without SCD [3–5], although two separate studies suggest that asthma may be more common among those with SCD compared to those without; [6, 7] 2) those who have SCD and asthma have higher rates of vaso-occlusive morbidity (pain and ACS episodes) compared to those with SCD and no asthma; [3, 5, 6, 8] and 3) asthma is a risk factor for mortality in SCD [9, 10].

Several questions exist with regards to these epidemiologic associations. For the question of asthma prevalence in SCD, there has been no population-based study of individuals with SCD compared to a comparable population without any underlying disease. The best available data are from prospective studies of those with SCD who are generally followed at academic tertiary care SCD centers and enrolled irrespective of symptoms. Data gleaned from these cohorts are not necessarily representative of the SCD population at large, making it difficult to truly understand the prevalence of asthma in this population. The Cooperative Study of SCD (CSSCD) was a longitudinal prospective cohort study which began enrolling patients regardless of symptoms or disease severity in 1978. Among 291 participants followed from infancy until at least age 5 years (mean length of follow up = 11 years) the prevalence of asthma was 17 % [5]. In addition to the CSSCD, the Sleep and Asthma Cohort (SAC) Study, began enrolling 253 children 4–19 years of age with sickle cell anemia (SCA, referring to those with HbSS and HbSβ° genotypes) in 2004 and followed them prospectively until 2014. The prevalence of asthma in the well-characterized SAC cohort is 28 % [11]. However, the asthma prevalence reported in SAC may not be truly representative of the general population with SCD, as children were enrolled from three tertiary care centers where clinicians may have had a particularly high index of suspicion for asthma [12]. The Pulmonary Hypertension and Hypoxic Response (PUSH) prospective cohort study enrolled 376 children age 3–20 years regardless of symptoms, and reported an asthma prevalence of 24 % [13]. One cross-sectional study conducted in Jamaica compared 80 children with SCD to a control group of 80 non SCD children found that 48 % of the SCD group had ever had “a touch of asthma” compared to 22 % of the control group [6].

Second, and perhaps more important, is how asthma is defined in these studies (Table 1). In the CSSCD cohort where associations between asthma and pain, ACS, and mortality were noted, asthma is defined as a personal or parental report of a physician diagnosis of asthma. Other studies that conclude asthma is a risk factor for morbidity use a similar definition, which is fairly standard for epidemiologic studies of asthma [14–16]. The PUSH study defined “asthma” as the presence of a report of “medical history of asthma or wheezing” without review of the medical record, [17] while SAC defined “asthma “ as a parent report of a physician diagnosis plus the use of a controller medication, confirmed by review of the medical record [3]. The big question that many of us are trying to answer is “What exactly IS asthma in SCD? How do we know it when we see it?” Part of the challenge for clinicians caring for SCD patients is that many of the common features of asthma – wheezing, airway obstruction, and even airway hyperresponsiveness during a bronchoprovocation challenge – are common in SCD but occur in isolation without other supporting evidence to warrant an asthma diagnosis. We explore these concepts further in the following sections.

**Table 1:** Summary of studies reporting prevalence of asthma in SCD patients

Study (year)	Study design	SCD type	Ages	How defined	Prevalence (%)
Boyd et al. (2006) [5]	CSSCD: 291 children enrolled in prospective cohort study	HbSS	Enrolled <6 months and followed through >5 years	Parent report of physician diagnosis of asthma	17 %
Rosen et al. (2014) [11]	SAC: 243 children in a prospective cohort study	HbSS or HbSβ^0^	4–19 years	Physician diagnosis of asthma and use of asthma medication ever, via parent report and confirmed with chart review [3]	28 %
Knight-Madden et al. (2005) [6]	Case control study of 80 children with SCD from Jamaica with 80 age, ethnic matched controls	HbSS	5–10 years	Parent report using modified ISAAC questionnaire (“touch of asthma”) for “lifetime asthma;” and wheeze or cough in past 12 months lasting 7 days in absence of URI for “current asthma”	48 % asthma ever, 41 % current asthma
Paul et al. (2013) [13]	PUSH: 503 children with SCD in a prospective cohort study	All types	3–20 years	Parent answers yes to question “medical history of asthma or wheezing” [17]	24 %
Cohen et al. (2011) [21]	114 adults with SCD in a prospective cohort study	All types	18–72 years	Positive response to the ATS-DLD question “Has a doctor ever diagnosed you with asthma” plus use of a controller medication	30 %

### Wheezing: common in SCD and independently associated with morbidity

Wheezing is a common clinical finding among SCD patients. Data from 1722 ACS episodes that occurred in the CSSCD cohort, demonstrated wheezing in 11 % of patients upon admission to the hospital, and was ultimately present in 26 % during the course of the ACS episode. Wheezing was equally common among children and adults [18]. In a second multicenter study of 538 patients with 671 episodes of ACS, wheezing was detected during 26 % of ACS episodes [19]. In a retrospective cohort study of 262 children with SCD who presented to an urban emergency department over a 4 year period, 19 % had at least one presentation in which wheezing was documented, although fewer than half of those children carried an asthma diagnosis [20]. In an unselected single center cohort of 114 adults, 30 % had at least two recurrent episodes of wheezing leading to shortness of breath, of whom almost half did not have a diagnosis of asthma [21]. Investigating which SCD patients with observed wheezing should have actually been diagnosed with asthma remains an area for future study.

Wheezing, without a co-existing diagnosis of asthma, has been associated with SCD morbidity in both children and adults. Among 159 children with SCA, DeBaun et al. found that a parental report of an episode of wheezing producing shortness of breath was associated with increased risk of a future episode of ACS (IRR 1.7, *p* = 0.04) during a median 5.2 years of follow-up [22]. In a prospective cohort of 262 patients with SCD (aged 6–68 years) seen in the Emergency Department, documentation of wheezing on physical exam (irrespective of asthma) was associated with more than two times the incidence of future vasoocclusive and ACS episodes during a 4 year follow-up period [20]. In the aforementioned mentioned study of 114 adults with SCD, while physician-diagnosed asthma was not associated with either increased rates of pain or ACS, 2 or more episodes of severe wheezing was associated with approximately a twofold increase of both vasoocclusive and ACS episodes as well as increased risk of early death (HR 4.2, 95 % CI 1.0–17.5, *p* < 0.05) [21]. Finally, in addition to prior studies showing respiratory symptoms in known asthmatics precede vaso-occlusive crises, [23, 24] recent evidence suggests a temporal relationship between respiratory symptoms including wheezing and SCD morbidity even in the absence of asthma. In a mostly adult SCD cohort (mean age 29 years) of 47 participants in whom asthma was *ruled out* via a prospective multistep algorithm that included participant report and medical record review, researchers conducted repeated telephone questionnaire interviews to ascertain the presence of cough or wheeze during consecutive 8 week periods over a mean of 9 months of follow-up. 68 % of participants reported at least one episode of cough or wheeze during the follow-up period, usually in the setting of colds; report of cough and/or wheeze symptoms during one 8 week period was associated with a two-fold increased rate of ED visits for pain in the subsequent 8 week period [25].

### Airway obstruction: common in SCD

Airway obstruction has been reported to be common among children with SCD, and has been demonstrated as early as infancy. Koumbourlis et al. performed infant pulmonary function tests with 20 SCD infants (including 12 with HbSS and 8 with mixed heterozygous hemoglobinopathies) and found elevated functional residual capacities (FRC), decreased maximum expiratory flows at FRC, and decreased time at maximum expiratory flows relative to the total expiratory time, suggesting hyperinflation and lower airway obstruction [26] Their group then studied 61 children age 5–18 years with HbSS and reported a 35 % prevalence of lower airway obstruction, [27] though their definition of an obstructive pattern (FEV_1_/FVC < 0.85, scooping of the flow/volume curve, and a “disproportionately low FEF_25–75_ % predicted”) [28] differs from the current ATS/ERS guidelines [29]. In the prospective, longitudinal PUSH study, 97 participants with HbSS or HbSβ° age 7–20 years completed spirometry and plethysmography for lung function classification. 18 children (19 %) met criteria for obstruction, defined as having an FEV_1_/FVC ratio and either an FEV_1_ or FEF2_5–75_ below the lower limit of normal. Forty-four percent of those with obstruction (*N* = 8) had a history of asthma or wheezing [17].

The significance of lower airway obstruction in SCD is not well understood. In a single institution, referred clinic cohort of 102 children with SCD, lower airway obstruction (defined by the standard ATS/ERS definition of an FEV_1_/FVC ratio <95 % CI for age, gender, and height [30]) was associated with twice the prospective rate of pain compared to those with normal lung function (IRR 2.16 (1.08–4.26); *P* = 0.027) [31]. A second retrospective chart review study of 127 patients concluded that a history of ACS events was associated with having lower airway obstruction on spirometry, but the definition of obstruction in that study was single cut-off for the FEV_1_/FVC ratio (85 %) rather than using the current ATS/ERS guidelines [32]. Further longitudinal research is needed to investigate associations between appropriately defined lower airway obstruction and SCD morbidity (i.e., do ACS events in childhood impact lung function in adulthoods? Is airway obstruction associated with future risk of SCD morbidity?).

### Airway hyperresponsiveness: common in SCD, but does it matter?

There are several small cross sectional studies that show a high prevalence of airway hyperresponsiveness in SCD (Table 2). In several small pediatric studies with sample sizes ranging from 21–40 children, airway hyperresponsiveness (AHR) to cold air or methacholine was present in 14–77 % of children with SCD [33–36]. In similar small studies of adults, prevalence of AHR ranged from 31–48 %, three to four fold higher than among control subjects in those studies [37, 38]. Given these small sample sizes, the Sleep and Asthma Cohort research group sought to establish the prevalence of AHR to methacholine in their unselected cohort of 243 children with SCA. When the 99th participant in the study (age = 14 years) was hospitalized for his first ever vaso-occlusive crisis following his methacholine challenge, [39] the SAC group removed methacholine challenge testing from their protocol. Among the 99 participants who had completed that methacholine testing, 55 had a PC_20_ < 4 mg/ml [40].

**Table 2:** Summary of studies addressing prevalence and clinical correlates of airway hyperresponsiveness in children and adults with SCD

Study (year)	Study design	Ages	Prevalence of AHR	Clinical Correlates of AHR
Leong et al. (1997) [34]	Cold air challenge in 32 children with SCD	6–19 years	67 % with 10 % drop in FEV_1_	No difference in prevalence of AHR to cold air between SCD patients with and without asthma;
Sylvester et al. (2007) [33]	Cold air challenge in 42 children with HbSS	6–16 years	14 % with 10 % drop in FEV_1_	No association between prior history of ACS and likelihood of having a positive cold air challenge test
Strunk et al. (2008) [35]	MCT in 21 clinically referred children with SCD and respiratory symptoms	5–18 years	67 % with PC_20_ < 12.5 mg/ml	Not evaluated
Ozbek et al. (2007) [36]	MCT in 31 children	6–16 years	77 % with PC_20_ < 16 mg/ml, 58 % with PC_20_ < 1 mg/ml	No differences in PC_20_ between those with and without history of ACS (*p* = 0.06)
Field et al. (2011) [40]	MCT in 99 children with HbSS or HbSβ^0^		55 % with PC_20_ < 4 mg/ml	Methacholine DRS not associated with STR, BDR, peripheral blood eosinophil count, or asthma diagnosis; DRS was associated with serum IgE and LDH
Chaudry et al. (2014) [41]	Cohort study 50 children with SCD and 50 non-SCD controls, 93 completed MCT	10–18 years	Prevalence of PC_20_ below a specified threshold not reported;	No difference in DRS between those DRS higher in children with asthma in both groups; No differences in DRS between HbSS and HbSC; DRS not associated with FEV_1_, FEV_1_/FVC, FeNO, or IGE among the SCD group;
Vendramini et al. (2006) [37]	MCT in 26 adults with and 28 controls without SCD	18–41 years	PC_20_ < 16 mg/ml in 31 % of SCD, 7 % of controls	Positive methacholine challenge not associated with having a baseline obstructive pattern on spirometry
Sen et al. (2009) [38]	MCT in 31 adults with SCD	18–44 years	48 % with PC_20_ < 16 mg/ml, 42 % with PC_20_ < 4 mg/ml	Significant association between the number of retrospective ACS events and having a positive methacholine challenge

*Abbreviations: ACS* acute chest syndrome, *BDR* bronchodilator responsiveness, *DRS* dose response slope, *MCT* methacholine challenge test, *PC*_*20*_ provocative concentration dose associated with a 20 % drop in FEV1; *SCD* sickle cell disease, *STR* skin test reactivity

The significance of the increased prevalence of airway reactivity to methacholine among SCD patients is not well understood. In a pulmonary function and biomarker study of 50 children with SCD and 50 non-SCD controls, Chaudry et al. found no difference in the methacholine dose response slope (DRS [defined as the % drop in FEV_1_ per increasing dose of methacholine]) between children with SCD versus controls, but did find that the DRS was higher among children with an asthma diagnosis than those without (4.46 vs 0.64, *p* = 0.006) regardless of their SCD status. That said, in multivariable models, DRS was not associated with FEV_1_, FEV_1_/FVC, fractional exhaled nitric oxide (FeNO) levels, or IgE among the SCD group [41]. Similarly, among the 99 children with SCA in SAC, the DRS was not associated with FEV_1_, FEV_1_/FVC, or FeNO, and was not associated with having an asthma diagnosis, bronchodilator responsiveness, having positive allergy skin tests, or the peripheral eosinophil count. DRS was, however, associated with increased serum IgE and lactate dehydrogenase (LDH) levels, reflective possibly of increased atopy and hemolysis respectively [40]. The association between methacholine DRS with LDH suggests a unique mechanism of AHR in SCD patients that may be more reflective of SCD pathogenesis than atopy.

Data are conflicting regarding whether airway responsiveness is independently associated with SCD morbidity. Chaudry found no differences in the DRS between those with the more severe HbSS and the milder HbSC genotypes [41]. In terms of whether prior history of ACS is associated with current AHR, Ozbek et al. found that the difference in PC_20_ results between those with and without a history of ACS did not reach significance (0.64 vs. 3.28 mg/ml, *p* = 0.06), though this may have been a function of sample size [36]. Sylvester et al. found no between-group differences in % drop in FEV_1_ after cold air challenge between those with and without ACS (median drop of 2.8 % in both groups), and that among the 6 children with a positive cold air challenge, none had a prior history of ACS [33]. In contrast, in a small adult cohort of 31 adults with SCD, there was a significant correlation between the number of retrospective ACS events and the methacholine PC_20_ [38].

### Other asthma features and SCD: markers of atopy and family history of asthma: their relationship to asthma and to SCD morbidity

Given the association between an asthma “diagnosis” and SCD morbidity juxtaposed with the frequency with which children with SCD have wheezing, airway obstruction, and/or airway hyperresponsiveness, investigators have started to investigate whether other factors that have been related to asthma in non-SCD children could help to diagnose asthma in SCD. Studies looking at whether IgE levels can be used to discriminate between those with and without asthma have been inconsistent. While some studies have demonstrated that children and adults with SCD and asthma have higher IgE levels than those without asthma, [21, 42, 43] Strunk et al. found that there was no difference in IgE levels between those with and without a physician diagnosis of asthma among children in the SAC cohort [3]. Having ≥ 2 positive skin tests to aeroallergens was more common among those with asthma compared to those without asthma in the SAC cohort, but skin test reactivity did not add much to a predictive model for discriminating “asthma” from “no asthma” in this cohort [3]. Ross et al. reported that SCD children with an asthma diagnosis were more likely to have positive ImmunoCAP testing to a 6-item food allergy panel than children without asthma (61 % vs. 35 %, *p* = 0.002) [43]. In terms of family history, a parental history of asthma was one of the most important features discriminating children with a physician diagnosis of asthma from children without asthma [3]. Data from 211 children in the CSSCD cohort demonstrated that having a first degree family relative with asthma was a significant risk factor for a personal history of asthma (odds ratio 6.4, 95 % CI 2.8 to 14.1, *p* < .001). While parental history of asthma was not associated with increased rates of pain or ACS, sibling history of asthma was independently associated with increased risk of VOC, even after adjusting for personal history of an asthma diagnosis (adjusted IRR 1.91, 95 % CI 1.18 to 3.09, *P* = 0.008) [44].

Atopic features have been associated with SCD morbidity. In a cohort of 80 Jamaican children with SCD, having at least one positive allergy skin prick test was associated with significantly increased odds of having “recurrent” ACS (≥2 episodes, OR 4.8, 95 % C.I. 1.5–16.1, *p* < 0.01) [6]. Among 521 children with SCD in the Silent Cerebral Infarct Trial, both an asthma diagnosis and elevated total serum IgE levels were independently associated with increased rates of ACS events [42]. DeBaun et al. found that having 2 or more positive allergy skin tests was an independent risk factor for future ACS events (IRR 1.87, 95 % CI 1.16-3.00, *p* = 0.01) [22].

### What is known about mechanisms of airway disease in SCD?

Animal models of SCD have an exaggerated inflammatory response. Nandedkar et al. compared mice expressing normal human (HbA) or normal murine (Wild type or WT) hemoglobin to transgenic SCD mice. Mice were sensitized to ovalbumin to induce lung inflammation, had a two week recovery period, and then received either high (“HiSen”) or low (“LoSen”) levels of an inhaled ovalbumin challenge daily for 10 days. 10 % of the SCD mice died during the recovery period prior to initiation of the inhalation challenges, compared to no deaths in the control mice groups. SCD LoSen mice had more airway eosinophilia than HbA or WT LoSen mice, and the SCD HiSen mice had higher IgE levels than WT or HbA mice suggesting increased inflammation. Furthermore, during the inhalation challenge period, 10 % of the LoSen and 30 % of the HiSen SCD mice died, compared with 4 % of the HbA mice and none of the WT mice, suggesting an impact of inflammation on SCD mortality [45]. In a second study, the same group found that chimeric SCD mice had a higher baseline leukocytosis and IL-13 levels compared with chimeric HbA mice. Following induction of experimental asthma by ovalbumin sensitization, chimeric SCD mice had dramatically increased IL-5 expression, greater airway resistance in the large and small airways, and greater airway responsiveness to methacholine compared to HbA mice, further demonstrating a greater baseline inflammatory profile and subsequent exaggerated airway inflammation among SCD mice [46]. In a more recent study, unsensitized SCD mice had increased perivascular inflammation and evidence of an increased Th2 response including increased total lymphocytes, CD8+ T cells, and CD4+ T cells (with increased total Treg cells) and increased levels of IL-5 when compared to control mice. SCD mice also had more robust responses to ovalbumin in terms of BAL leukocytosis and eosinophilia, higher levels of ovalbumin-specific serum IgE levels, and increased expression of IL-6 and 13 compared to control mice. Interestingly and in contrast to the previously described study, [46] ovalbumin-sensitized SCD mice had less airway hyperresponsiveness to methacholine than did control mice despite the observed enhanced inflammatory response. The authors hypothesize that this finding could indicate that airway obstruction may not be due to methacholine-induced bronchoconstriction (and thus, less responsive to conventional asthma therapies) but due to alternative SCD-specific mechanisms [47].

Not all studies support the notion that there is an increased Th2 response in SCD. Knight-Madden and colleagues compared cytokine profiles in 80 children with SCD in Jamaica and 53 children with SCD in the United Kingdom to cytokine profiles among local healthy controls. Children with SCD in Jamaica were more likely to have asthma and more likely to have increased Th2 cytokines (defined as detectable IL-4/undetectable IFN- γ) than local Jamaican age- and ethnically- matched controls while children in the UK with SCD were less likely to have asthma and increased Th2 cytokines than local controls [48]. This discrepancy was attributed to tropical increases in helminth exposure among the Jamaican children, which promotes a Th2 inflammatory response that may have been particularly exaggerated among the children with SCD [48].

Exhaled nitric oxide (eNO) levels have been examined in patients with SCD. In the general population and in cohorts of children with asthma, eNO has been used as a marker of airway inflammation– and has been associated with several features of atopic asthma such as total serum IgE, peripheral blood eosinophilia, and the FEV1/FVC ratio in children with asthma [49, 50]. Studies have demonstrated eNO levels in children with SCD to be lower, [51] higher, [52] or the same [41, 53] as eNO levels in healthy controls. Chaudry et al. compared features of airway function in 50 children with SCD to 50 healthy controls. They found that eNO levels were higher in atopic children compared to those without atopy across both the SCD and control groups, but eNO was not correlated with the degree of airway responsiveness to methacholine among the SCD group [41]. Whether or not eNO measurements can be used as a tool to aide in deciding which children with SCD do or do not have asthma has not been thoroughly investigated. Data from methods of measuring airway inflammation, such as measuring the degree of eosinophilia or neutrophilia from induced sputum, are also lacking.

More recently, investigators have emphasized the role of vascular factors on obstructive airway phenotypes in SCD. In a British study of 25 children with HbSS and 25 age- and ethnic- matched controls, the children with SCD had greater pulmonary capillary blood volume and increased respiratory system resistance (R_5_ % predicted, as measured by impulse oscillometry) compared to controls. Among the children with HbSS only, pulmonary capillary blood volume was positively correlated with R_5_ and the ratio of residual volume/total lung capacity (RV/TLC), and negatively correlated with FEV_1_ and mean maximal expiratory flow (MMEF _25–75_) suggesting that airway obstruction in SCD may be related to increased cardiac output and increased pulmonary blood volume in response to chronic anemia [54]. The same group of investigators obtained high resolution computed tomography (HRCT), echocardiograms, and pulmonary function tests in 35 adults with SCD and found associations between increased cardiac output on echocardiography, increased pulmonary artery/bronchus ratios on HRCT, increased respiratory system resistance, increased RV/TLC, and decreased FEV_1_ and FEF_25–75_ supporting the notion that abnormalities in pulmonary vascular volumes have effects on airway function in patients with SCD which are independent of asthma [55].

### How do we put it all together?

Wheezing is common in SCD, and may or may not be associated with a diagnosis of asthma in this population. Airway obstruction and airway reactivity are also common but are also poor indicators of asthma in SCD. Both wheezing and pulmonary function test evidence of airway abnormalities may be important markers of SCD severity. The most recent version of the NHLBI guidelines on the Evidence-Based Management of Sickle Cell Disease make the following recommendations: 1) assess for signs and symptoms of respiratory problems by history and physical examination; 2) in patients with signs or symptoms of respiratory problems, further assessment (including pulmonary function testing) is recommended; and 3) in otherwise asymptomatic individuals, screening pulmonary function testing is NOT recommended [56]. The recommendation against screening all individuals for abnormal pulmonary function is based on the paucity of evidence that pulmonary function screening offers clinical benefit or is cost-effective. To date, there have been no published studies regarding interventions targeted towards individuals with abnormal lung function.

We recommend the following for all patients with SCD:

1. Careful symptom screening with a high index of suspicion for: history of wheezing, atopy, family history of asthma, and any history of dyspnea or exercise limitation.
2. Any patient positive for the above historical features should be referred for evaluation with a pulmonologist including pulmonary function testing.
3. Consider allergy testing if symptoms suggest that sensitization to environmental exposures could be exacerbating symptoms.
4. Optimization of SCD management in consultation with a hematologist including consideration of hydroxyurea, currently the only FDA-approved therapy for the management of SCD which reduces morbidity and mortality in these patients.
5. In patients for whom a diagnosis of persistent asthma seems appropriate based on history (repeated episodes of wheezing and cough in response to known triggers, clinical response to bronchodilators, and other historical features consistent with asthma including personal and family history of atopy and first degree relative with asthma), treatment with anti-inflammatory controller therapy according to the NHLBI guidelines is recommended [3, 57].

## Conclusions and future directions

Wheezing and airway function abnormalities are common in SCD, but as is true in the general population, “all that wheezes is not asthma.” We first need a better understanding of the mechanisms underlying these common clinical features so that we can differentiate asthma from what Knight-Madden and Greenough have termed “Recurrent Wheezing in Sickle Cell Disease (RWIS)” [58]. Once we have a better understanding of the mechanisms underlying airway abnormalities in SCD we can begin to explore the impact of therapeutic interventions on recurrent wheezing, airway obstruction, and/or asthma on short and long term SCD outcomes. This is an important, understudied area that warrants further investigation in an attempt to reduce morbidity and mortality in these patients.

## Abbreviations

ACS: Acute chest syndrome
AHR: Airway hyperresponsiveness
ATS/ERS: American Thoracic Society/European Respiratory Society
CSSCD: Cooperative Study of Sickle Cell Disease
DRS: Methacholine dose response slope
FeNO: Fractional exhaled nitric oxide
FEF_25–75_: Average forced expiratory flow during the middle 25-75 % of a forced expiratory maneuver
FEV_1_: Forced expiratory volume in one second
FVC: Forced vital capacity
LDH: Lactate dehydrogenase
PC_20_: Provocative concentration dose associated with a 20 % drop in FEV_1_
PUSH: Pulmonary Hypertension and Hypoxic Response study
SAC: Sleep and Asthma Cohort study
SCA: Sickle cell anemia
SCD: Sickle cell disease
